# The Effects of Social Feedback Through the “Like” Feature on Brain Activity: A Systematic Review

**DOI:** 10.3390/healthcare13010089

**Published:** 2025-01-06

**Authors:** Artemisa R. Dores, Miguel Peixoto, Carina Fernandes, António Marques, Fernando Barbosa

**Affiliations:** 1 Psychosocial Rehabilitation Laboratory, Center for Rehabilitation Research (LabRP-CIR), Escola Superior de Saúde, Instituto Politécnico do Porto, 4200-072 Porto, Portugalajmarques@ess.ipp.pt (A.M.); 2 Laboratory of Neuropsychophysiology, Faculty of Psychology and Education Sciences, University of Porto, 4200-135 Porto, Portugal; carinafernandes@ufp.edu.pt (C.F.); fbarbosa@fpce.up.pt (F.B.); 3 Faculty of Human and Social Sciences, University Fernando Pessoa, 4249-004 Porto, Portugal; 4 Molecular Oncology and Viral Pathology Group, Research Center of IPO (CI-IPOP) & RISE@CI-IPOP (Health Research Network), Portuguese Oncology Institute of Porto (IPO Porto)/Porto Comprehensive Cancer Center (Porto.CCC), 4200-072 Porto, Portugal

**Keywords:** social media, like button, EEG, fMRI

## Abstract

Background: Problematic social media (SM) use is a growing concern, particularly among adolescents who are drawn to these platforms for social interactions important to their age group. SM dependence is characterized by excessive, uncontrolled usage that impairs personal, social, and professional aspects. Despite the ongoing debate over recognizing SM addiction as a distinct diagnostic category, the impact of social feedback, particularly through the “like” button, on brain activity remains under scrutiny. Objective: This systematic review aims to study the neural correlates of online social feedback, focusing on the effects of the “like” feedback on brain activity using fMRI and EEG. Methods: The review followed the recommendations of the Preferred Reporting Items for Systematic Reviews and Meta-Analysis Protocols (PRISMA). Results: The review included 11 studies with 504 participants, identifying key brain structures such as the amygdala, ventromedial prefrontal cortex (vmPFC), and ventral striatum involved in reward processing. Positive feedback (“likes”) activates areas like the nucleus accumbens (NACC), vmPFC, and amygdala, with NACC correlating with increased SM use intensity. Negative feedback activates the ventrolateral prefrontal cortex (vlPFC) and left medial prefrontal cortex (mPFC). Behavioral data indicates that positive feedback influences subsequent social interactions. Conclusions: The review highlights disparities in the literature regarding the neural response to social feedback, emphasizing the need for further research to clarify the roles of sex, personality traits, and the person giving feedback. Overall, understanding the neurobiological underpinnings of SM engagement is essential for developing effective interventions to prevent or address the negative effects of excessive SM use.

## 1. Introduction

Problematic use of social media (SM) platforms is an ever-growing concern that affects various populations, especially people from low-income countries [1], with moderate to low school achievement, low parental control [2], low self-esteem [3], feelings of loneliness, fear of negative evaluation, and behavioral inhibition [4]. Adolescents seem to be amongst the more vulnerable populations due to SMs’ unique appeal to their age range. They are drawn to these platforms for social interactions [5]. However, social feedback plays a critical role in shaping social behavior and well-being throughout life, from childhood to older adulthood. In early development, positive social feedback from peers and caregivers helps foster social skills, self-esteem, and a sense of belonging. As individuals age, social feedback continues to influence behavior, reinforcing social bonds and maintaining relationships. In older adults, social feedback becomes increasingly important due to the reduction in social networks, leading to potential feelings of loneliness and isolation (e.g., Tragantzopoulou & Giannouli, [6]). Positive social interactions and feedback can mitigate these effects by promoting emotional well-being and reducing feelings of loneliness, while the absence or negative social feedback may exacerbate loneliness and contribute to mental health decline in this population.

SM dependence is defined as the excessive and uncontrolled usage of SMs, leading to impairments in personal, social, and professional aspects [7,8]. Excessive users of SM can show abstinence symptoms, such as anxiety [9,10], stress, and depression [9,11]. They can also develop a tolerance, resulting in the need to progressively increase the time spent on SMs to obtain the same level of gratification experienced in the early stages. Another relevant aspect of dependence is the excessive worry associated with SM-related behaviors, and a lack of self-control with internet usage [7]. However, other results show no negative outcomes, only showcasing some specific behaviors as potentially dangerous [12]. One of the reasons for this discrepancy is that SM dependence could also be explained based on the person’s preferred SM, as some results show that changing behaviors could be more difficult in certain SM [13] Given these differences, this type of addiction is still being debated in the literature, with the Diagnostic and Statistical Manual of Mental Disorders (DSM-5; [14]) and International Classification Diseases (ICD-11; [15]) not recognizing addiction to SM as a separate diagnostic category, despite it being studied as a behavioral addiction when it becomes excessive and affects the daily life of an individual [8,16].

In the context of SM, the “like” button acts as a reward mechanism, providing quick and simple feedback on users’ social media activity [17]. Given its purpose, the “like” feature can motivate users to adjust their publications and online behaviors to maximize the chances of receiving this reward [18].

The Interaction of Person-Affect-Cognition-Execution (I-PACE) model [19] offers insights into the underlying mechanisms involved in the development of various behavioral addictions. The I-PACE model highlights the importance of the interaction between predisposing factors, affective and cognitive responses to stimuli, and executive functions—such as inhibitory control and decision making—in the development of behavioral addiction. In the case of social media, like gambling or gaming disorders, the process may involve heightened cue-reactivity and craving, coupled with diminished inhibitory control, which can contribute to habitual behaviors. Further studies are needed to explore both the common and distinct mechanisms involved in addiction, obsessive–compulsive disorders, impulse control disorders, and substance use disorders, as these conditions share underlying neurobiological processes.

Studies have used functional magnetic resonance imaging (fMRI), electroencephalography (EEG), and evoked-related potentials (ERP) to better understand the effects of social feedback through the “like” on brain activity. Each technique offers advantages and limitations. fMRI has excellent spatial resolution, making it possible to identify the brain regions involved in the perception and processing of “likes” [20]. On the other hand, EEG, due to its high temporal resolution, is especially suitable for studying rapid cognitive processes such as attention and sensory responses.

fMRI studies show that the amygdala, ventromedial prefrontal cortex (vmPFC), and ventral striatum are key structures in reward processing [21,22,23]. The vmPFC plays a key role in this network through the observation and evaluation of the reward (i.e., “number of likes”; followers), motivating behavior towards it. The amygdala further solidifies the behavior-oriented toward the positive outcome, which can lead to SM addiction. Additionally, using SM may be associated with reduced amygdala volume [22]. The striatum, which shares various connections with different brain structures, allows the integration of information from various modalities and is necessary for learning and evaluating rewards [21,23], which in turn supports motivated behavior [23]. The involvement of the striatum in reward processing extends to social situations, where certain situations, such as a compliment from a colleague, result in increased activity [21].

For EEG data, evoked potentials, specifically the P300 and N200 components, have been used to investigate online social interactions. The P300 is associated with the allocation of attention and processing of relevant stimuli [24,25,26]. In the context of social networks, an increase in the amplitude of the P300 suggests a greater allocation of neuronal resources to the processing of these socially relevant stimuli. The N200 is often associated with conflict detection and decision making [27].

Current research shows the activation of several structures related to reward processing and the impact that their activation has on continued engagement with SM. As such, the need for research that further validates the effects of certain aspects, such as the “like” feature, on continued engagement is increasingly present. Despite some online social interactions having similarities with offline interactions, they are not identical. For instance, reward processing differs, as online social rewards lead to higher activation of structures such as the nucleus accumbens (NACC) while showing less amygdala recruitment [22]. Identifying these differences, we aim to systematically review studies on the neural correlates of online social feedback, using EEG and fMRI as investigative techniques. The main aim is to systematize the neuronal correlates and neurobiological processes that underlie the response to social feedback, focusing on how this stimulus is processed by the brain structures involved in social reward processing. Considering the relevance of investigating the “like” feature of social media platforms through fMRI and EEG (e.g., Meshi et al. [28]; Bhanji & Delgado [21]), the following are the proposed main research questions:

Research question 1: What are the effects of the “like” feedback on neuronal regions associated with reward processing, assessed through fMRI?

Research question 2: What are the effects of the “like” feedback on brain activity, assessed through EEG?

Additional questions involve exploring potential differences in brain activity associated with feedback valence (i.e., positive versus negative) and comparing habitual versus sporadic social media users. Behavioral studies on this topic will also be reviewed.

## 2. Method

The recommendations of Preferred Systematic Review and Meta-analysis (PRISMA; [29]) were followed to guide the general stages and protocols of this review. The PRISMA 2020 Checklist can be found in Appendix A.

### 2.1. Search Strategy

This review was performed according to the actualized Preferred Reporting Items for Systematic Reviews and Meta-Analyses (PRISMA) guidelines [29]. Articles published until September 2024 were selected from PubMed, Web of Science, and EBSCOhost (including the Academic Search Complete, Psychology and Behavioral Sciences Collection, CINAHL Plus with Full Text, Fonte Acadêmica, MedicLatina, PsycARTICLES, PsycBOOKS, and PsycINFO databases).

The search expression was as follows: “(“like feedback” OR “like symbol” OR “social media feedback” OR “social feedback”) AND (fMRI OR “functional magnetic resonance imaging” OR EEG OR electroencephalography OR “Event-related Potentials” OR ERP) AND (“reward processing” OR “reward system” OR “brain regions” OR “neural regions” OR “ventral striatum” OR “nucleus accumbens” OR “dopaminergic pathways” OR “reward circuitry”) NOT (marketing OR “fake news”)”. To prevent publication and source selection bias, an additional manual search was performed.

### 2.2. Study Selection

We included observational cross-sectional studies that investigated the neural processing of social media-related feedback by samples of adolescents and young adults (12–35 years old). After being included in reporting research on the topic of the review, articles were excluded according to the following criteria: (a) articles without a group of adolescents or young adults processing social media-related feedback (criterion 1: other population); (b) articles that did not investigate the neural processing of social media-related feedback (criterion 2: other measures); (c) inaccessible articles or articles without information about the neural processing of social media-related feedback (criterion 3: lack of data); (d) articles published in other languages than English (criterion 4: inaccessible language); and (e) reviews, commentaries, case series, or methods (criterion 5: wrong publication type). Articles reporting only duplicated data were also excluded, and when articles reported an expansion of previously conducted research, data from the most recent article were selected (criterion 6: duplicated data).

### 2.3. Screening and Selection of Records

The results of the literature search were compiled on Rayyan QCRI [30]. Following Higgins and Green’s [31] guidelines, after the elimination of duplicates, two researchers blindly screened the titles and abstracts, excluded the articles that were out of topic, and retained the remaining studies. When this task was completed, the screening was unblinded. The reference list of the included empirical studies and reviews were also screened, retaining titles in the topic that did not appear in the systematic search. Two authors read all the retained studies and, independently, decided to include or exclude them. Disagreements in both stages were solved by consensus.

### 2.4. Quality Assessment

Two independent reviewers assessed the quality of the included studies using the Appraisal Tool for Cross-Sectional Studies (AXIS), a recent tool developed to assess the methodological quality of observational cross-sectional studies [32]. AXIS contains 20 questions regarding the introduction, methods, results, and discussion of each study. Each question could be answered with “yes” (1 point) or “no”/“don’t know” (0 point). Disagreements were solved by consensus.

### 2.5. Data Collection and Analysis

The data of each included article were added to an extraction sheet developed for this review and refined when necessary. When available, the following variables were extracted from each article: year of publication; sample size (including males and females); mean age and standard deviation; neural imaging method; goals of the study, self-report measures used; details of the experimental task; and neurophysiological and behavioral results.

## 3. Results

### 3.1. Search Results

**Figure 1:**
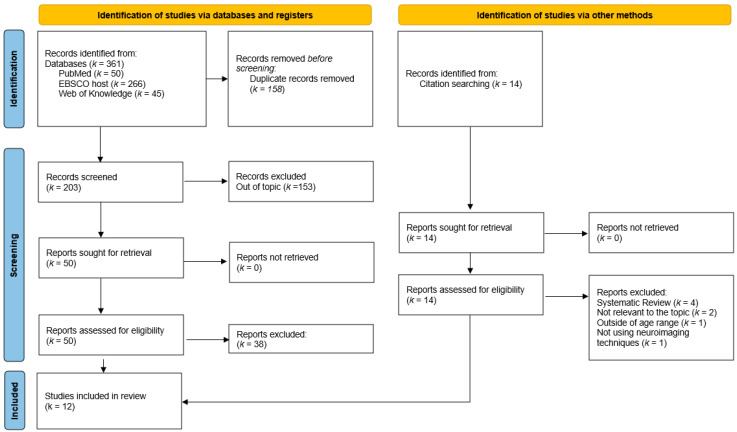
PRISMA flow diagram.

A total of 203 articles were identified through the search string. Initially, 158 articles were excluded as duplicates. A total of 50 articles were then analyzed based on their titles and abstracts. Of these, 38 articles were excluded due to the following criteria: (a) out of topic (*n* = 32); (b) other age range (*n* = 2); (c) lack neuroimaging techniques (*n* = 2); (d) wrong publication type (*n* = 2). Twelve articles were selected for full-text analysis, of which five studies were included. An additional 14 articles were hand-searched, of which seven were excluded due to the following criteria: (a) systematic review (*n* = 4); (b) not relevant to the topic (*n* = 1); (c) outside of the age range (*n* = 1); and (d) not using neuroimaging techniques (*n* = 1). Seven articles were selected for a full-text analysis from this batch, of which six studies were included. In total, 12 articles were included in the final review. Cohen’s kappa was used to assess inter-rater agreement, with a score of 0.93 indicating substantial agreement [33]. The study selection process is illustrated in the PRISMA flow diagram (Figure 1).

### 3.2. Studies Characteristics

The reviewed studies included a total of 537 participants, with 195 males and 295 females. One study did not report the participants’ sex [34]. The techniques used to assess the participants were fMRI (*n* = 9); EEG (*n* = 3); and PET scan (*n* = 1). Regarding questionnaires, four studies [17,34,35,36] did not report using any questionnaire. The remaining studies [25,28,37,38,39,40,41] used a variety of instruments to assess their variables, with no questionnaire being consistently used across the literature. The methods used in the reviewed studies are presented in Table 1.

The assessment of the studies using the AXIS (Appendix A) revealed that scores varied between 14 and 18 (*M =* 15.8; *SD* = 1.17). Several common weaknesses were observed across the majority of the studies. A significant issue was the lack of sample size justification, as most studies did not provide a clear rationale for their chosen sample sizes. This limitation impacts the statistical power of their conclusions and raises concerns about the reliability of their results. Additionally, power calculations were not explicitly reported in any of the studies, further weakening the robustness of the findings. Another recurring problem was the failure to identify potential biases. Few studies adequately discussed or accounted for biases that might influence their results, which is crucial for ensuring the accurate interpretation of the data. Furthermore, while most studies demonstrated strengths in terms of replicability, only a few addressed the generalizability of their results beyond the specific populations they studied, limiting the broader applicability of their findings. Despite these weaknesses, the studies generally excelled in terms of the clarity of their objectives, the appropriateness of their study designs, and the adequate presentation of results, reflecting the overall high standards in these areas.

### 3.3. Neurophysiological and Behavioral Results

Regarding the neurophysiological results (Table 2), several studies investigated the effect of social rewards/rejection with the SM context through both positive (i.e., “like”) and negative feedback (i.e., negative comment, “dislike”). The structures sensitive to social feedback, regardless of valence, are the striatum [28,39], thalamus, cerebellum [39], ventral anterior cingulate cortex (ACC) [34], ventrolateral prefrontal cortex (vlPFC), medial prefrontal cortex (mPFC), occipital cortex, and superior temporal gyrus [41]. Specifically, for receiving a “like”, the striatum, thalamus, ventral tegmental area (VTA), mPFC, motor cortex, occipital cortex, and cerebellum are activated [17].

Some studies also focus on specific activation towards positive and negative feedback. For positive feedback, the results showed the activation of the left posterior insula, medial superior parietal lobe, precuneus [41], NACC, ventral midbrain, vmPFC, mid-cingulate cortex, amygdala [35], and dorsal, ventral, and retrosplenial posterior cingulate cortex (PCC) [35,41]. Furthermore, the NACC activation to positive feedback was positively correlated with SM use intensity [28]. For negative feedback, the results showed the activation of the vlPFC and left mPFC [41]. Feedback can also come in the form of many or few “likes”, where many “likes” are shown to activate the mPFC, lateral occipital cortex, hippocampus, NACC, caudate, putamen, thalamus, and VTA [36]. In terms of EEG activity, receiving many or few “likes” showed a predominance of beta waves, while receiving a moderate amount of “likes” showed a predominance of alpha waves [38].

The person giving the feedback can also influence brain activity through social feedback. A person more positively viewed led to higher activity in the amygdala and vmPFC [35]. Sex also influences brain activity, specifically, receiving positive feedback from the opposite sex results in higher activation of the right orbitofrontal cortex (OFC) and right anterior insula [35]. Expectations also play a role in understanding the neurological activity elicited by feedback. Positive feedback consistent with expectations activates the ventral mPFC, right subcallosal cortex, right PCC, left OFC, caudate nucleus, left precuneus, and left thalamus in contrast to unexpected negative feedback. Negative feedback consistent with expectation activates the right subcallosal cortex, left caudate, right putamen, right middle frontal gyrus, and right inferior frontal gyrus in contrast to unexpected positive feedback. Age, resistance to peer pressure, and social anxiety levels are also shown to influence these results [37]. A narcissistic personality trait also influences brain activity, with lower P3 amplitudes when receiving many “likes” on a selfie [25].

The reported behavioral results (Table 2) corroborate neuroimaging data, showing that positive feedback was more desirable [39]. When it comes to giving a “like”, sex can influence the motivation to do so, as female faces are rated as more positive, which in turn makes them more rewarding to view, because higher-rated faces are reported to be more rewarding [35]. This is consistent with the results showing that women receive more “likes” per photo [41].

## 4. Discussion

This systematic review aimed to analyze neurophysiological studies (i.e., studies using fMRI and EEG methodologies) investigating online social reward processing, mainly the feedback provided through the “like” feature.

The review found significant variability in the objectives, methods, and results across the studies. The objectives often align with key social factors influencing social media (SM) use, such as social rewards [13,43], usually in the form of a “like”, reward learning, and decision making [23].

Regarding the main research questions—what are the effects of the “like” feedback on neuronal regions associated with reward processing, assessed through fMRI and through EEG?—the analysis of the data related to receiving feedback, both positive and negative, revealed activation in several brain structures associated with reward processing, including the striatum and thalamus [28,39]. The structures involved in goal-oriented and social behavior, such as the ventrolateral prefrontal cortex (vlPFC) and medial prefrontal cortex (mPFC) [44,45], are also engaged in feedback processing. These findings align with the existing literature that underscores the role of these structures in social reward processing [21,22,46,47].

Differential brain activity for positive versus negative feedback was also identified. The nucleus accumbens (NACC) appears particularly crucial for engagement with SM, as its activation correlates with positive feedback and the intensity of SM use [28]. This supports the notion that the NACC plays a significant role in motivating behavior related to positive social gains and avoiding social punishment [48]. The insula is involved in reward anticipation and avoiding social punishment [46], a finding corroborated by the reviewed data [41].

The amygdala’s activation in response to positive feedback is expected [35], given its role in social behavior coordination [49] and positive outcome processing [22]. In contrast, the results for negative feedback were less extensive, primarily showing activation in the vlPFC and mPFC [41]. This gap highlights the need for future research into the neuroanatomical areas involved in processing negative feedback. Broader research on social rewards has identified the NACC, insula, and right inferior frontal gyrus as sensitive to negative social outcomes [46,48,50].

Specifically, when it comes to receiving a “like”, the striatum, thalamus, hippocampus, and VTA are activated [17]. This is consistent with their roles in various aspects of social rewards and behavior [21,23,46,51]. Notably, the insula, amygdala, ventromedial prefrontal cortex (vmPFC), and paracingulate cortex are uniquely activated in response to receiving a “like” rather than giving feedback [18]. However, it is worth noting that some expected structures, such as the NACC, which is typically involved in processing positive feedback [28], were not identified in this study [17].

The significance of the “like” feature as social feedback is further supported by studies examining the effects of the quantity of “likes” on brain activity. Similar activation patterns were observed in structures such as the mPFC, NACC, and thalamus [36]. Additional structures activated by receiving many “likes” include the caudate, brain stem, VTA, and hippocampus [36]. The VTA and hippocampus are associated with memory and pro-social behavior [47,51,52] and contextual memory for social rewards [53], respectively. The caudate and brain stem are also integral to models of social reward processing [46], with the caudate being involved in both monetary and social rewards [54]. Despite their roles in social behavior, these regions were not consistently reported, with some appearing only in specific paradigms related to feedback valence or quantity of positive feedback (i.e., number of “likes”). Understanding these discrepancies is crucial for elucidating the impact of social feedback on SM.

Furthermore, EEG data revealed predominantly beta wave activity in response to receiving many “likes” [38]. Given beta waves’ involvement in decision making and selective attention [55], EEG findings corroborate fMRI data, highlighting the activating effect of numerous “likes”.

Overall, the results show considerable variability in effects, and while not contradictory, they do not establish a consistent activation system for online social rewards. Methodological differences and various influencing factors, such as the characteristics of the feedback giver and recipient, including sex and personality traits, can affect reactions to positive feedback. For example, a more positive rating from the feedback giver was associated with the amygdala and vmPFC [35]. This suggests that higher social hierarchy may involve greater amygdala activation due to its role in hierarchical learning and reward processing [56]. The vmPFC’s role in processing social feedback and self-relevant information [57] might explain its activation when receiving feedback from individuals with higher ratings. Additional structures, such as the hippocampus and mPFC, also relate to social hierarchy [56], indicating a need for further research into their involvement in feedback processing from high-status individuals. While evidence on sex differences in processing social feedback is limited, one study indicated increased activity in the right OFC and right anterior insula in response to feedback from the opposite sex [35]. The particular activation of these structures could be related to somatic activation [58] or sexually relevant stimuli, such as attraction toward the opposite sex [59]. Personality traits may also influence SM engagement through rewards tailored to specific traits, affecting brain activity such as the P3 component [25].

Non-neuroimaging studies reinforce the importance of the characteristics of the giver and recipient of the “like”. On one hand, the characteristics of the giver are shown to be more relevant in determining the subjective value of the “like” than the number of “likes” received [60]. On the other, the characteristics of the receiver (i.e., age) seem to alter the perceived value of the “like” [61]. Personality is one of these characteristics, with certain personality profiles, such as narcissism [62], leading to higher SM use [63,64], further modulated by sex and age [65]. Indeed, these individual characteristics seem to be potential vulnerabilities towards a pathological engagement with SM, as several studies have shown that variables such as impulsivity, narcissism, fear of missing out, and low self-esteem can be associated with an increased risk of developing this kind of involvement [66]. As such, and as pointed out in some studies [35] the neural correlates of social feedback could vary based on the characteristics of the giver and recipient.

The findings from our systematic review offer valuable insights into the neural correlates of social media feedback, particularly the “like” feature, and its impact on brain activity.

One of the key clinical insights is the role of brain structures such as the nucleus accumbens (NACC), vmPFC, and amygdala in reward processing. The activation of these regions in response to positive social feedback highlights the neurobiological underpinnings of social media use, which can inform clinical approaches to addressing problematic social media behavior, particularly in adolescents. The excessive activation of these reward-related brain areas, especially the NACC, is linked to increased intensity of social media use, which may contribute to the development of dependency or addiction-like behaviors [28]. This suggests that interventions aimed at reducing social media use could benefit from targeting the reward systems and enhancing self-regulation strategies to counterbalance excessive reward-seeking behaviors.

The review highlights the amygdala’s role in social behavior and its involvement in processing positive feedback. Given that the amygdala is also associated with emotional regulation, its activation in the context of social media may explain why some individuals are more prone to anxiety or mood disturbances in response to online interactions [22]. Clinically, this underscores the need for mental health interventions that address the emotional impact of online social interactions, particularly in populations vulnerable to anxiety or depression.

Additionally, the findings suggest a significant difference in how positive and negative feedback is processed in the brain. The relatively limited involvement of brain structures in response to negative feedback, particularly when compared to the robust activation seen with positive feedback, may indicate that users of social media, particularly adolescents, are more sensitive to social rewards than to social punishments. Clinically, this raises concerns about how the continuous pursuit of social validation may reinforce maladaptive behaviors, such as excessive engagement with social media, while potentially diminishing resilience to social rejection or negative feedback [41].

There are similarities between the brain structures involved in processing the “like” feedback and the structures involved in processing gambling outcomes. For example, the activation of the amygdala and the ventromedial prefrontal cortex (vmPFC) to gambling outcomes could be an indicator of problematic gambling [67,68]. Non-neuroimaging data corroborates the similarities between conditions, as evidenced by increased decay of the reward value over time (delay discounting) [69,70], or by problematic engagement possibly serving as a coping mechanism with adverse consequences [19,71]. Furthermore, individuals with more severe SM-related problematic behavior are more likely to exhibit additional problematic behaviors or even develop a behavioral addiction, such as gambling disorder [72]. Despite the similarities, a “like” functions as a social reward, in contrast to the monetary reward associated with gambling outcomes. One key difference lies in the value of a “like”, which is influenced by the characteristics of the giver. However, to our knowledge, no studies have directly compared these two types of rewards to clarify their differences. In light of these findings, future research should explore the longitudinal effects of social media engagement on mental health, especially in individuals with pre-existing vulnerabilities such as low self-esteem or social anxiety. Understanding how these neural patterns evolve could inform prevention strategies for adolescents and young adults at risk of developing dependency on social media platforms.

Clinically, the evidence from our review calls for the development of therapeutic interventions aimed at moderating the neural reward systems activated by social feedback. Cognitive–behavioral interventions focusing on self-regulation, emotional resilience, and the management of online interactions could help mitigate the potential negative impacts on mental health. Additionally, educational programs for adolescents, focusing on the psychological mechanisms behind social media usage, may empower them to navigate online spaces more consciously and healthily.

Despite the contribution of this study to the literature, some limitations must be considered when interpreting the results. One of the main limitations of this systematic review is the heterogeneity of methodologies across the included studies. The included studies used different neuroimaging techniques, including fMRI and EEG, each of which has distinct strengths and limitations. For instance, fMRI provides excellent spatial resolution, allowing the identification of specific brain regions involved in reward processing, but it lacks the temporal resolution needed to capture the fast dynamics of brain activity. On the other hand, EEG offers high temporal resolution but lacks spatial specificity, making it difficult to identify the exact brain regions responsible for certain neural responses. This variation in neuroimaging methods hinders the direct comparison of findings across studies and introduces potential biases related to the limitations inherent to each technique.

Additionally, there was significant variability in the participant demographics across the studies, particularly regarding age, sex, and social media usage habits. Most of the studies included adolescents and young adults, but the age ranges varied, and some studies did not report detailed demographic data, such as sex distribution. This heterogeneity can influence the results, as neural responses to social feedback may differ by demographic differences, as well as personality traits or susceptibility to social influence. As a result, it is difficult to generalize the findings to a broader population or to make definitive conclusions about how different groups may respond to social media feedback. Also, the studies had relatively small sample sizes, which may affect the statistical power of their findings. These limitations make it difficult to synthesize the results and draw comprehensive conclusions. Overcoming these limitations in future research will be crucial for gaining a more complete understanding of how social media feedback influences brain activity.

## 5. Conclusions

The current findings on the neural correlates of online social feedback, focusing on the effects of the “like” feedback on brain activity, using EEG and fMRI, show a fragmented picture, with various findings that, while not contradictory, do not clarify a distinct network of brain structures involved in processing feedback, such as the “like” button. To better understand this, it is crucial to consider a wider range of influencing variables, including sex and personality traits, which have been shown to affect brain activity. Additionally, peripheral physiological indicators could offer valuable insights into users’ reactions to SM and their subsequent behaviors.

It is important to note that giving or receiving a “like” involves different brain processes, and our review focused solely on the experience of receiving a “like”. This limitation means that our findings might not fully capture the nuances observed in studies examining both giving and receiving “likes”. Another limitation arises from some studies using alternative forms of positive feedback instead of the standard “like” button, which makes difficult the systematic analysis of results.

To enhance the validity of current findings, the increased use of EEG, due to its superior temporal resolution, would be beneficial. Comparing excessive SM users with regular or healthy users could also provide more precise insights into group differences.

In summary, as SM becomes increasingly integrated into daily life, more research is needed to explore the individual and SM characteristics, namely the role of the “like” feedback that drives user engagement. It is crucial to examine a wide range of variables to ensure that research remains relevant in the rapidly evolving field of social media, and the “like” feedback should be among them. Robust evidence is necessary to support and refine the theoretical models of online social rewards and to develop effective prevention and intervention strategies.

## Figures and Tables

**Table 1:** Summary of the methodology used to assess online feedback.

Authors	Methods						
	Participants (*N*)	Gender (F/M)	Age (*M ± SD*) Min—Max	Neural Imaging Methods	Study Objectives	Self-Report Measures	Experimental Task
Somerville et al. [34]	42	Not reported	Not reported	fMRI	Testing social rejection and expectancy violation.	Not reported	Participants were initially photographed to have their picture rated by another; the rating was manipulated. They would view unfamiliar faces and had to say (yes or no) if they would like them based on first impressions. A second question was asked if the person in the photo would like the participant (yes or no). The same faces would appear after some time, with some having the opposite of the feedback initially given to them by the participant.
Izuma et al. [39]	19	F = 10 M = 9	21.6 ± 1.5	fMRI Pet scan	Testing if a good reputation activates reward-based circuits, and if these circuits are the same as those for monetary rewards.	Social Desirability Scale; Impressions Management Scale; Rosenberg Self-esteem Scale (RSES); NEO Five-Factor Inventory.	Participants would talk about a prevalent topic, talk about themselves, and take pictures. This material would then be rated by another group. In the second phase, predetermined feedback (positive or negative) was given.
Davey et al. [35]	19	F = 12 M = 7	19 ± 2.9 15–24	fMRI	Testing the activation of reward-related regions to receive a “like”. Testing the effects of positive feedback and being “liked” by the opposite gender on certain brain regions.	Not reported	Participants had their pictures taken to be rated by another; the rating was manipulated. The participants thought they would view a set of neutral faces of other participants, which were from a pre-existing database. They would classify these faces based on the likelihood of liking the other person, and afterward, the faces appeared associated with a rating, supposedly given to the participant by the person in the photo.
Gunther et al. [37]	Total: 57 Prepubertal children: 12 Early adolescents: 14 Older adolescents: 15 Young adults: 16	Prepubertal children: F = 7 M = 5 Early adolescents: F = 8 M = 6 Older adolescents: F = 7 M = 8 Young adults: F = 8 M = 8	Prepubertal children: 9.7 ± 0.9 8–10 Early adolescents: 13.3 ± 0.8 12–14 Older adolescents: 17.1 ± 0.6 16–17 Young adults: 21.7 ± 1.9 19–25	fMRI	Testing the neural correlates of social acceptance and rejection in relation to age.	Raven’s Progressive Matrices; Resistance to Peer Influence Questionnaire; Multidimensional Anxiety Scale for Children; Self-perception Profile for Children; Self-perception Profile for Adolescents.	Participants viewed unfamiliar age-matched faces and had to say (yes or no) if the person in the photo would like them based on first impressions. The same faces would appear after some time, with half having the opposite of the feedback initially given to them by the participant.
Meshi et al., 2013 [28]	31	F = 17 M = 14	23.1 ± 3.2	fMRI	Testing if differences in the activation of NACC between social gains for the self and for others predicts SM use.	Facebook Intensity Scale; Structured Interview; RSES; Reynolds Social Desirability Scale-C; Narcissistic Personality Inventory; Mehrabian Conformity Scale; Beck Depression Inventory-II.	Participants saw their picture and a photo of another person and had to correctly identify whose picture it was. After answering, a word would appear that would correspond to a third-party assessment of the picture, this was manipulated. The feedback given was either positive or neutral.
Oumeziane et al. [42]	33	F = 19 M = 14	M = 26.34, SD = 6.25	EEG/ERP	To compare the neural processing of social (likes) and monetary rewards	Not reported	In the monetary task, participants anticipate and react to monetary rewards or punishments by responding quickly to specific cues, typically involving a delay period between stimulus and response. The social version of the task is similar, but the participants received likes instead of money.
Sherman [36]	32	F = 18 M = 14	13–18	fMRI	Testing the influence of “likes” and photos depicting risky behaviors on areas responsible for cognitive control.	Not reported	Initial submission of Instagram photos by participants. While in the fMRI, they viewed neutral images, images with risky and non-risky behaviors, and images submitted by other participants. The photos were displayed with either a high or low number of “likes”. Half of the participants’ photos had a high number of “likes”, while the other half had few “likes”. In the second version, the photos with a high number of “likes” had a lower number, and vice versa. When viewing the photos, the participants had to either “like” or skip them.
Sherman, Hernandez et al. [17]	58	F = 34 M = 24	18.2 13–21	fMRI	Testing the neural correlates of giving and receiving a “like”.	Not reported	Initial submission of Instagram photos by participants. The participants viewed neutral images, images with risky behaviors, and images submitted by the participants, and had to either “like” or skip them.
Sherman, Greenfield et al. [40]	Total: 58 High schoolers: 32 University students: 26	F = 35 M = 23	High schoolers: 16.8 ± 1.4 University students: 19.9 ± 1.1 Overall 13–21	fMRI	Testing the neural effects of receiving many “likes”. Testing age-related differences in the neural activation of reward processing and executive function.	Revised Cognitive Appraisal of Risky Events.	Initial submission of Instagram photos by participants. The participants viewed the “likes” on their pictures given by others, with “likes” being manipulated. While in the fMRI, they viewed neutral images, images with risky behaviors, and images submitted by participants. Half of the participants’ photos had a high number of “likes”, while the other half had few “likes”. In the second version, the photos with a high number of “likes” had a lower number, and vice versa.
Nash et al. [25]	77	F = 59 M = 18	20.8 ± 3.73	EEG	Testing the correlation between narcissistic traits and the fulfillment of narcissistic needs with social network engagement.	40-item Narcissistic Personality Inventory.	Participants used their phones with the Instagram app. The participants experienced social exclusion by performing a Cyberball task. The participants then performed a startle task, after which they were assigned to one of three conditions. For the selfies with likes condition, the participants posted a selfie with popular hashtags and were able to see the “likes” obtained in real-time; this was manipulated. For the selfie-only condition, the participants uploaded a selfie, and then viewed the picture for 5 min with no feedback. The neutral picture condition only stared at a picture of gravel.
Issa and Jabbouri [38]	19	F = 14 M = 5	19–23	EEG ECG	Testing neurophysiological responses to “likes”.	Sociodemographic Questionnaire.	Participants were divided into three conditions: frequently receiving “likes”; less frequently “likes”; and receiving a moderate number of “likes”. The participants sent 30 recently posted pictures of themselves. These pictures were mixed with pictures from other participants. They would then have to “like” or “skip”. Throughout the experiment, several controlled notifications were sent with the number of “likes” received and the participants’ positions on a scale.
Wikman et al. [41]	Total: 92 Sample 1: 26 Sample 2: 66	Sample 1: F = 10 M = 16 Sample 2: F = 42 M = 24	Total: 18.7 ± 0.72 17–20	fMRI	Replicating previous findings on neural activation with virtual social interactions. Testing the relation between brain activity towards peer feedback and SM use.	Open-ended questionnaire about social network use.	Participants posted opinions on a Facebook group, receiving feedback from other participants. They were told that the feedback was prerecorded authentic feedback to the posted opinions. The participants were exposed to several controversial opinions and based on their answer (agree or disagree) a post in line with their answer was shared. The response given by peers, was either positive or negative, with some neutral reactions. For the control condition a neutral, factually correct statement was presented; the participant would then say true or false. The responses to the comment would also be neutral. The statement would appear for 3 s, followed by a 3 s window to answer, and by the post for 3 s.

Notes. Social media platform (SM); female (F); male (M); functional magnetic resonance imaging (fMRI); Electroencephalogram (EEG); Electrocardiogram (ECG); Electrooculogram (EOG).

**Table 2:** Summary of the neurophysiological and behavioral results.

Authors	Methodology		Results	
	Participants (*N*)	Technique	Neurophysiological	Behavioral
Somerville et al. [34]	42	fMRI	Dorsal ACC: sensitive to expectation violation Ventral ACC: sensitive to feedback valence (positive/negative)	-
Izuma et al. [39]	19	fMRI	Striatum, thalamus, and cerebellum: activation for both social and monetary rewards Caudate nucleus and putamen: similar activation for monetary and social rewards	Trials with positive feedback were more desirable.
Davey et al. [35]	19	fMRI	NACC, ventral midbrain, vmPFC, mid-cingulate cortex, amygdala, dorsal, ventral, and retrosplenial PCC: activation for positive feedback Amygdala, and vmPFC: higher activation for positive feedback received from a person with a higher rating Right OFC and right anterior insula: sensitive to gender	Female faces were rated as more positive than male faces, and faces rated higher were more rewarding to view.
Gunther et al. [37]	57	fMRI	Ventral mPFC, striatum, left subcallosal cortex, ACC, MCC, right putamen, right amygdala, left hippocampus, and right parahippocampal gyrus: activation for expectation violation, in adults Ventral mPFC, right subcallosal cortex, right PCC, left OFC, caudate nucleus, left precuneus, and left thalamus: higher activation for expected positive feedback compared to unexpected negative feedback Right subcallosal cortex, left caudate, right putamen, right middle frontal gyrus, and right inferior frontal gyrus: higher activation for expected negative feedback compared to unexpected positive feedback	Young adults chose “yes” more often than prepubertal children and early adolescents and were faster to respond than prepubertal children.
Meshi et al., 2013 [28]	31	fMRI	Ventral striatum: activation for both social and monetary rewards Left NACC: activation to positive feedback, correlated with Facebook usage intensity	-
Sherman [36]	32	fMRI	Visual cortex, and cerebellum: higher activation for neutral photos with many “likes” Left frontal cortex, precentral, middle frontal, and inferior frontal gyrus: higher activation for risky photos with many “likes” Precuneus, mPFC, lateral occipital cortex, hippocampus, NACC, caudate, putamen, thalamus, ventral tegmental area, and brain stem: higher activation for self-photos with many “likes” NACC: higher activation for photos with high amounts of “likes”, except risky photos	Participants were more likely to “like” photos liked by others, and to not “like” unpopular photos.
Oumeziane et al. [42]	33	EEG/ERPs	Social and monetary rewards elicit comparable ERP latencies and scalp topographies across several processing stages (reward cue, outcome anticipation, and outcome evaluation), highlighting the possibility of a common neural network.	-
Sherman, Hernandez et al. [17]	58	fMRI	Striatum, thalamus, VTA, mPFC, motor cortex, occipital cortex, and cerebellum: activation for receiving many “likes”	Neutral images were more likely to be “liked” than pictures depicting faces. Appeal, being funny, and being similar to a picture taken by the participant were the more frequent motives for liking a picture. A visceral reaction was a more common reason to give a “like”.
Sherman, Greenfield et al. [40]	58	fMRI	NACC: activation for self-photos with many “likes”	Popular images received more “likes” than unpopular ones. This effect was more prominent for self-pictures.
Nash et al. [25]	77	EEG	P3: decreased amplitude on a selfie with “likes” compared to only “selfie” and the neutral condition, in people with high narcissism and higher scores of leadership/authority; increased amplitude for a selfie with “likes” compared to the neutral condition, in narcissistic people with low scores of leadership/authority	-
Issa and Jabbouri [38]	19	EEG ECG	Beta wave: predominant when receiving many or few “likes” Alpha waves: predominant when receiving moderate/average number of “likes” ECG: similar activity when receiving many or few “likes”	-
Wikman et al. [41]	92	fMRI	vlPFC, mPFC, occipital cortex, and superior temporal gyrus: activation for positive and negative feedback vlPFC; anterior insula; and left mPFC: activation for negative feedback Left posterior insula, medial superior parietal lobe, precuneus, PCC, and superior frontal gyrus: activation for positive feedback	Female participants spent more time on social media and received more “likes” per photo.

Notes. Anterior cingulate cortex (ACC); nucleus accumbens (NACC); ventromedial prefrontal cortex (vmPFC); posterior cingulate cortex (PCC); orbitofrontal cortex (OFC); ventral tegmental area (VTA); medial prefrontal cortex (mPFC); social media platform (SM); middle cingulate cortex (MCC); ventrolateral prefrontal cortex (vlPFC); Appraisal tool for Cross-Sectional Studies (AXIS).

## Appendix A

**Table A1:** Appraisal Tool for Cross-Sectional Studies (AXIS).

	Study											
AXIS	1	2	3	4	5	6	7	8	9	10	11	12
Introduction												
1	1	1	1	1	1	1	1	1	1	1	1	1
Methods												
2	1	1	1	1	1	1	1	1	1	1	1	1
3	0	0	0	0	0	0	0	0	0	0	0	1
4	1	1	1	1	1	1	1	1	1	1	1	1
5	0	0	0	0	0	0	0	0	0	0	0	1
6	0	0	0	0	0	0	0	0	0	0	0	0
7	1	1	1	1	1	1	1	1	1	1	1	1
8	0	0	0	0	0	0	0	0	0	0	0	0
9	1	1	1	1	1	1	1	1	1	1	1	1
10	1	1	1	1	1	1	1	1	1	1	1	1
11	1	1	1	1	1	1	1	1	1	1	1	1
Results												
12	1	1	0	0	1	1	1	1	1	1	0	1
13	1	1	1	1	1	1	1	1	1	1	1	1
14	1	1	1	1	1	1	1	1	1	1	1	1
15	1	1	1	1	1	1	1	1	1	1	1	1
16	1	1	1	1	1	1	1	1	1	1	1	1
Discussion												
17	1	1	1	1	1	1	1	1	1	1	1	1
18	1	1	0	0	1	1	1	1	1	1	0	1
Other												
19	1	1	1	1	1	1	1	1	1	1	1	1
20	1	1	1	1	1	1	1	1	1	1	1	1
Total	16	16	14	14	16	16	16	16	16	16	14	18
